# Efficacy and safety of Chinese herbal medicine granules plus chemotherapy in patients with EGFR-mutated advanced lung adenocarcinoma post-progression on first-line EGFR-TKI: study protocol for a multicenter, double-blind, randomized controlled trial

**DOI:** 10.1186/s12906-025-05037-z

**Published:** 2025-11-19

**Authors:** Yizhao Du, Jialin Yao, Xuanting Ye, Guanjin Wu, Lingzi Su, Wenxiao Yang, Lijing Jiao, Yabin Gong, Ling Xu

**Affiliations:** 1 Department of Oncology, Yueyang Hospital of Integrated Traditional Chinese and Western Medicine, Shanghai University of Traditional Chinese Medicine, Shanghai, 200437 P.R. China; 2 Institute of Translational Cancer Research for Integrated Chinese and Western Medicine, Yueyang Hospital of Integrated Traditional Chinese and Western Medicine, Shanghai University of Traditional Chinese Medicine, Shanghai, 200437 P.R. China

**Keywords:** EGFR resistance, Advanced lung adenocarcinoma, Chemotherapy, Chinese herbal medicine, Randomized controlled trial

## Abstract

**Background:**

Lung cancer remains one of the leading causes of mortality globally. For patients with epidermal growth factor receptor (EGFR) -mutated lung adenocarcinoma who develop resistance to first-line EGFR-tyrosine kinase inhibitors (EGFR-TKIs), subsequent treatments typically involve a regimen that includes pemetrexed and platinum. However, the progression-free survival (PFS) achieved with this approach is limited to 4–5 months. There is an urgent need for effective and safe adjuvant therapies. Preliminary research suggests that Chinese herbal medicine (CHM) granules may extend PFS and improve quality of life (QoL), potentially offering a therapeutic strategy for advanced non-small-cell lung cancer (NSCLC). A rigorous, randomized clinical trial is warranted to confirm these findings. The aim of this study is to evaluate the efficacy and safety of CHM granules in combination with chemotherapy for patients with EGFR-mutated advanced lung adenocarcinoma following resistance to first-line EGFR-TKIs.

**Method/design:**

This study is a multicenter, double-blind, randomized, placebo-controlled clinical trial in China. A total of 244 patients will be enrolled and randomly assigned to either the treatment group (chemotherapy plus CHM granules, *n* = 122) or the control group (chemotherapy plus placebo granules, *n* = 122). The primary outcome measure is PFS, with secondary outcomes including overall survival (OS), objective response rate (ORR), disease control rate (DCR), patient-reported quality of life assessed by the Functional Assessment of Cancer Therapy-Lung (FACT-L) and Lung Cancer Symptom Scale (LCSS), and Traditional Chinese Medicine (TCM) clinical syndrome scores. Adverse events will also be closely monitored.

**Discussion:**

The study’s findings will provide an evidence-based evaluation of the combination of CHM granules with chemotherapy for treating EGFR-mutated advanced lung adenocarcinoma with resistance to first-line EGFR-TKIs. The results may inform the development of clinical guidelines for adjuvant therapy in this patient population.

**Trial registration:**

http://www.chictr.org.cn. Trial number: ChiCTR2000029144, Registered on 19 Aug, 2020.

**Supplementary Information:**

The online version contains supplementary material available at 10.1186/s12906-025-05037-z.

## Background

Lung cancer remains one of the leading causes of cancer-related deaths worldwide with a high mortality rate of 18%, ranking it among the leading types of cancer [1]. In China, the incidence of the disease is also on the rise annually, with an estimated 870,982 new cases and 766,898 deaths predicted for the year 2022 [2]. Approximately 70% of patients with non-small-cell lung cancer (NSCLC) are diagnosed at advanced stages, underscoring the urgency for effective treatments options [3]. With a deeper understanding of the molecular biological characteristics of lung cancer, precision therapies targeting various specific mutations are beginning to bear fruit. Targeted therapy has become one of the most important systemic treatment options for patients with advanced NSCLC.

Mutations in the epidermal growth factor receptor (EGFR) gene are established therapeutic targets within lung cancer treatment paradigms, with an overall somatic mutation rate of 30.6% in the Chinese NSCLC population [4, 5]. The advent of EGFR-tyrosine kinase inhibitors (EGFR-TKIs) marks a significant advancement in targeted therapy, offering superior efficacy in prolonging patient survival and reducing side effects compared to conventional chemotherapy [6]. The ongoing refinement of targeted therapeutics has led to a progressive enhancement in clinical outcomes. Notably, the FLAURA study demonstrated that third-generation EGFR-TKI, Osimertinib, significantly prolonged progression-free survival (PFS) compared to first-generation EGFR-TKIs (18.9 months vs. 10.2 months, HR = 0.46) [4].

Despite the initial therapeutic effectiveness, drug resistance remains an inevitable issue. The mechanisms of resistance to EGFR-TKIs are increasingly recognized and significantly limit the long-term efficacy. The resistance mechanisms are classified into two distinct categories [7]: EGFR-dependent, where alterations within the EGFR gene or pathway are observed, and the C797S mutation has been found to be the most common [8]; and EGFR-independent, which encompasses the development of bypass pathways, histologic transformation, and oncogenic fusions [9–11]. Targeted therapies aimed at resistance targets, such as MET amplification, HER2 amplification, PIK3CA mutations, BRAF mutations, and RAS mutations, are still in the clinical research phase and are not yet maturely recommended for widespread clinical practice [12]. The heterogeneity of lung cancer presents a significant challenge to personalized treatment approaches. Consequently, platinum-based chemotherapy, with or without bevacizumab, remains the standard treatment for advanced non-squamous NSCLC [13, 14]. However, the clinical outcomes following resistance to first-line EGFR-TKIs have been unsatisfactory. The IMPRESS study showed that the PFS for patients treated with cisplatin and pemetrexed post first-line EGFR-TKI resistance was a mere 5.4 months [15]. Additionally, the AVAPERL study indicated that for patients treated with a combination of bevacizumab, cisplatin, and pemetrexed only extended to 7.4 months, an additional 2 months [16]. Furthermore, treatment with pemetrexed, carboplatin, and bevacizumab, followed by maintenance therapy with pemetrexed and bevacizumab, resulted in numerous drug-related grade 3 or 4 adverse events (AEs), including anemia (14.5%), thrombocytopenia (23.3%), and fatigue (10.9%) [17], all of which significantly diminished patient quality of life. In light of these limitations, there is a growing interest in complementary medicine, such as Chinese herbal medicine (CHM), which has shown potential benefits in the treatment of NSCLC both clinically and experimentally.

In recent years, the application of traditional Chinese medicine (TCM) as an adjuvant therapy for NSCLC patients undergoing chemotherapy has been increasingly recognized. A systematic review and meta-analysis [18], encompassing 14 randomized controlled trials, including 1451 patients diagnosed with 1451 patients with advanced NSCLC, stages III-IV, demonstrated that patients treated with a combination of Chinese herbal medicine (CHM) and platinum-based chemotherapy had superior objective response rates (*p* < 0.001), disease control rates (*p* < 0.001), and improved quality of life (*p* = 0.014). Feiping Granule, originally named Yiqi-Yangyin-Jiedu Granules, were developed based on the “Fuzheng Treatment of Cancer” theory and have been clinically applied to enhance the therapeutic efficacy for patients with advanced NSCLC. Previous research has shown that the combination of maintenance chemotherapy and Feiping Granule can extend the PFS to 6.43 months, which is 2.7 months longer than that of the control group [19], highlighting the potential benefits of Feiping Granule in prolonging PFS. Additionally, previous research has demonstrated that Feiping Granule, when combined with adjuvant chemotherapy, can alleviate the side effects of chemotherapy and improve the quality of life [20]. Furthermore, scientific evidence has shown that Feiping Granule enhances the expression of EGR1 and exerts synergistic effects on lung cancer cell apoptosis [21], suggesting the potential of CHM to augment the efficacy of chemotherapy. In addition to being used in combination with chemotherapy, Feiping Granule, when combined with EGFR-TKIs, has also been shown to delay the onset of drug resistance and prolong PFS [22]. However, there remains an urgent need for a high-quality, large-sample, randomized controlled trial to establish the efficacy and safety of CHM as an adjuvant therapy in combination with chemotherapy for NSCLC patients who have developed resistance to EGFR-TKIs.

Therefore, this study aims to systematically evaluate the clinical efficacy and safety of CHM granules combined with chemotherapy for the treatment of stage IIIB/IV NSCLC patients who have developed resistance to EGFR-TKIs.

## Methods/ design

**Fig. 1:**
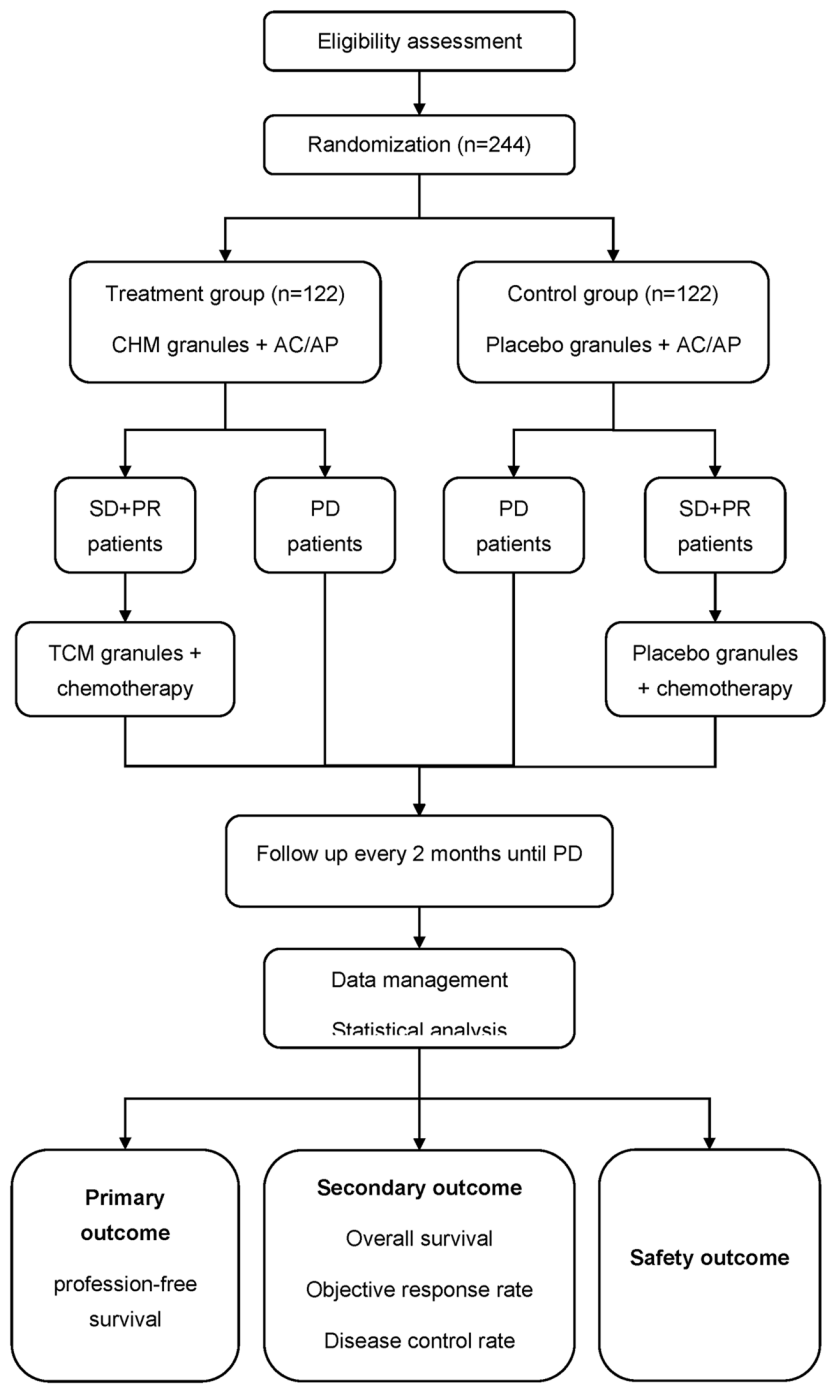
Flowchart of the study

This is a randomized, controlled, double-blind, multicenter clinical trial. This trial was registered with the Chinese Clinical Trials registry ChiCTR2000029144. This protocol was developed in accordance with the Standard Protocol Items: Recommendations for Interventional Trials (SPIRIT) 2013 statement [23] and its extension to TCM [24]. A total of 244 participants will be randomly assigned to the treatment group or the control group. Both groups will receive standard chemotherapy (carboplatin and pemetrexed, or cisplatin and pemetrexed, 4–6 cycles), with or without bevacizumab. Without disease progression, both groups will transition to continuation maintenance with pemetrexed, potentially including bevacizumab, continuing until disease advances. Each chemotherapy cycle involves the treatment group taking CHM granules: Hewei Granule for days 1 to 7 and Feiping Granule for days 8 to 21 post-chemotherapy, while the control group takes placebo granules daily for 21 days. Participants will be followed up every two months until disease progression or death to monitor tumor progression and survival time. The study flowchart is shown in Fig. 1. The schedule of enrolment, interventions, and assessments are shown in Table 1.

**Table 1:**
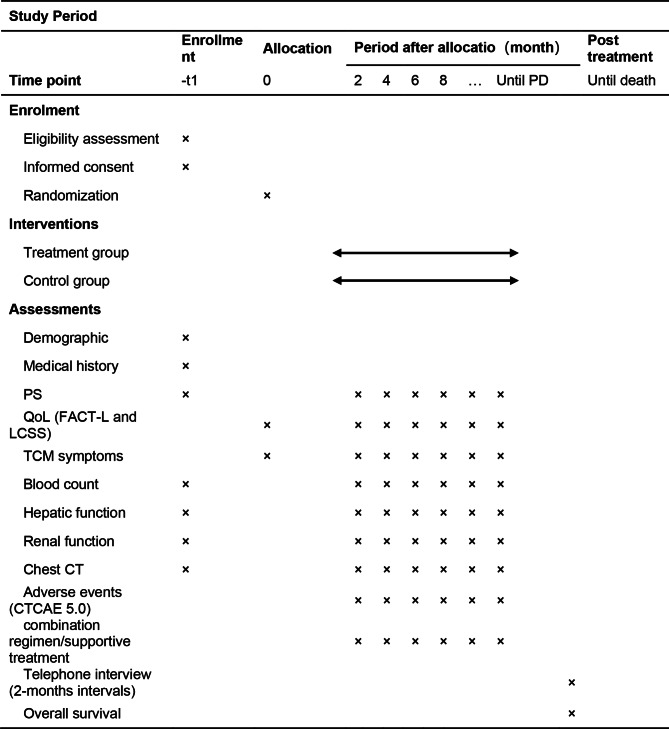
Schedule of enrollment, intervention, and assessment

### Ethical issues and informed consent

The study protocol (version 2.0 in December 2020) has been approved by the Research Ethics Committee of Yueyang Hospital of Integrated Traditional Chinese and Western Medicine, Shanghai University of Traditional Chinese Medicine (NO.2020-078). Prior to enrollment, participants will be thoroughly introduced to the detailed procedures of the study by the researchers. All issues and concerns related to the study will be specified. Subjects will also be informed of the possible benefits and potential risks to ensure that their participation in the study is entirely voluntary. Subjects who meet all of the inclusion criteria and none of the exclusion criteria will be enrolled after providing written informed consent.

### Sample size

In previous studies, the PFS of gefitinib combined with chemotherapy in the treatment of stage IIIB-IV EGFR mutation-positive advanced lung cancer was 4.5–5.7 months [15], the follow-up time of TCM combined with chemotherapy maintenance therapy was 4.18 months, and the PFS was 3.27–11.8 months [19]. In this study, PFS is the primary endpoint and it is conducted at α = 0.05 and β = 0.20. It is estimated that the PFS is 5.7 months in the control group and 8.4 months in the study group. The length of recruitment is 12 months and the total length is 24 months. The sample allocation ratio is 1:1, with 101 cases in each group. The dropout rate is 20%, calculated using PASS 14.0 software, with 122 cases in each group and a total sample size of 244.

### Study setting

Participants will be recruited through clinic and inpatient wards in 4 sites, Yueyang Hospital of Integrated Traditional Chinese and Western Medicine, Shanghai University of Traditional Chinese Medicine, Shanghai, China; Shanghai Pulmonary Hospital, Tongji University School of Medicine, Shanghai, China; Shanghai chest Hospital, Jiaotong University, Shanghai, China, and Fudan University Shanghai Cancer Center, in Shanghai, China.

### Randomization

Eligible patients enrolled at each site will be randomly allocated to either the chemotherapy CHM group or the chemotherapyplacebo group at a 1:1 ratio through a dynamic random method. When patient gender (male vs. female), age (65 years old vs. 65 years old), clinical stage (b, a, b), EGFR mutation site (19del, 21L858R), ECOG PS (Eastern Cooperative Oncology Group performance status) score (1, 2) and enrollment centre is input as stratified factors, the software will automatically output the results of randomization. Personnel for drug administration will be able to obtain a random number and group allocation immediately in the form of a short message service, and the entire process is confidential. The statistician generated the allocation sequence. Investigators will continue enrollment by screening eligible patients based on inclusion and exclusion criteria and assigning participants to interventions.

### Blinding

The trial is a double-blinded design, in which the randomization results and blind code will be kept strictly confidential until all intervention measures are allocated, registration, follow-up, data collection, data cleaning and analysis are completed. Participants and researchers, including medical staff, investigators, result evaluators and statisticians, will not know the distribution. Only if a SAE is relevant to the research medication, the assignment code will be broken. The date and reason for breaking the blinding code should be recorded in the case report form (CRF). Relevant institutions, including clinical research institutions, should be informed accordingly within 24 h.

### Recruitment

The recruitment of the study will use promotion posters, leaflets, digital media and specialist’s referral. People who are interested in the trial can contact the researchers by telephone. Researchers will conduct preliminary screening on the phone and invite these potential candidates to come to the clinics to have a further screening face to face. Chinese medicine practitioners will be responsible for conducting the screening and explaining the details of the study, such as content, arrangement, and possible risks etc. The eligible participants will be invited to join the study after signing the informed consent form.

### Inclusion criteria

(1) Patients with pathologically confirmed EGFR-sensitive mutations in stage IIIB-IV lung adenocarcinoma, without chemotherapy (Health Commission of PRC and National Health Commission of the People’s Republic of China, 2019).

1) Disease progression after treatment with first- or second-generation EGFR-TKIs and no mutation in T790M on the second biopsy.

2) Disease progression after treatment with third-generation EGFR-TKIs.

(2) Cisplatin/ Carboplatin + Pemetrexed with or without bevacizumab will be used for the treatment of the posterior line.

(3) Aged 18 to 74 years old.

(4) Eastern Cooperative Oncology Group performance status (ECOG PS) score 0–2, expected survival ≥ 3 months.

(5) No major organ dysfunction, blood routine, liver, kidney, heart function meet haemoglobin ≥ 100 g/L, absolute neutrophil count (ANC) ≥ 1.5 × 10^9/L, platelets ≥ 80 × 10^9/L, bilirubin ≤ 1.5ULN, alkaline phosphatase (AP), aspartate aminotransferase (AST) and alanine aminotransferase (ALT) ≤ 2.5 × ULN; INR ≤ 1.5 and creatinine ≤ 1.5ULN.

(6) Signed informed consent and agreed to participate in this project.

*EGFR sensitive mutation: Amplification refractory mutation system (ARMS) detection is preferred, and NGS detection can also be adopted. For EGFR-TKI-resistant patients, it is recommended to carry out secondary EGFR T790M detection (ARMS method, kit). When tissue samples are unavailable or insufficient for gene detection, both of them can be detected by T790M in peripheral blood.

### Exclusion criteria

(1) History of other tumours within 5 years.

(2) Patients with T790M who plan to use EGFR-TKI third-generation drugs for second-line treatment or meet the requirements for second-line treatment with other targeted therapies and immunotherapy.

(3) Patients have oligo-progression or central nervous system (CNS) progression and plan to continue EGFR-TKI therapy and local treatment.

(4) Patients with a history of radiotherapy in the past 2 months and are ready to receive radiotherapy.

(5) Symptomatic brain metastases.

(6) Previous or ongoing participation in other clinical investigators.

(7) History of cardiovascular disease: congestive heart failure > NYHA functional class II; Patients with unstable angina (angina symptoms at rest) or newly developed angina (starting in the past 3 months) or myocardial infarction occurring in the past 6 months. Active infection, Grade > 2 AEs (CTCAE. Version 5.0).

(8) Pregnant or lactating patients.

(9) Patients with a history of uncontrolled psychosis.

### Drop-out criteria

(1) Poor subject compliance during the trial, and insufficient data, affect the efficacy and safety evaluators.

(2) Serious adverse event (SAE), complications and special physiological changes occur, and it is not suitable to accept the continuation of the trial.

(3) Self-withdrawal during the trial.

(4) Combination of drugs, especially the combination of drugs that have a greater impact on the experimental drugs, affecting the efficacy and safety evaluation.

(5) Cases who withdrew from the trial, are lost to follow-up or died due to various other reasons before the course of treatment.

(6) Individual cases that are broken in blind studies: Patients with serious adverse reactions or serious complications during treatment; Patients who are unwilling to cooperate or unable to complete treatment.

### Interventions

**Fig. 2:**
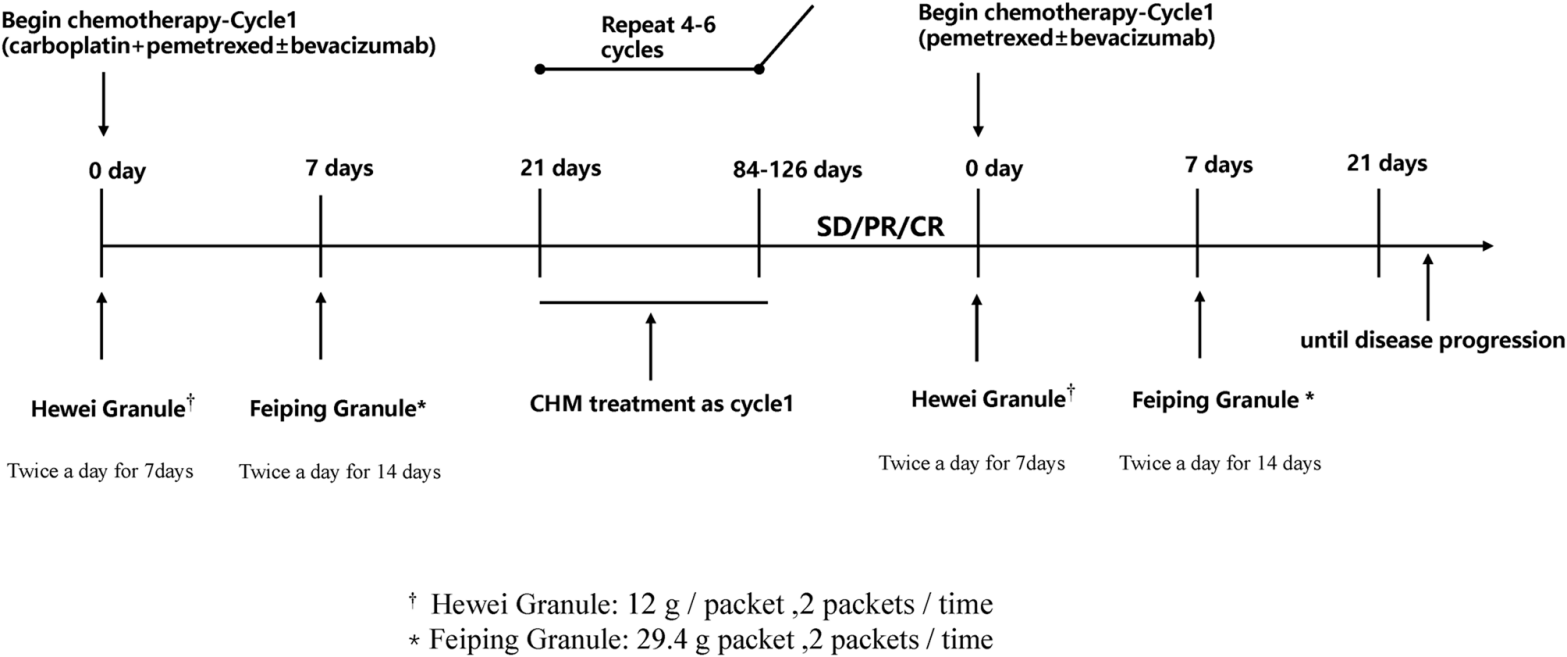
Specific process

Participants will be allowed to continue their routine medications except for Chinese patent drugs throughout the trial. Participants in both groups will receive the standard Western treatment regimen, which is defined as receiving intravenous doublet chemotherapy with carboplatin/cisplatin plus pemetrexed (AC/AP) for 4–6 cycles, to which bevacizumab (Bev) can optionally be added to the treatment regimen. If efficacy evaluation according to Response Evaluation Criteria in Solid Tumours (RECIST) version 1.1 shows complete response (CR), partial response (PR), or stable disease (SD), chemotherapy of pemetrexed (A) will be used until disease progression or the occurrence of unacceptable toxicity. Hewei Granule and Feiping Granule or placebo will be given daily on the first day after chemotherapy until the end of chemotherapy. Figure 2 will show the detailed intervention process. Participants will be stratified into two groups:

Treatment group: Chemotherapy + CHM Granules.

Control group: Chemotherapy + Placebo.

### Western medicine treatment

Patients should receive carboplatin (area under the curve, 4–5, ivgtt 30 min) or cisplatin (75 mg/m2, ivgtt 2 h) plus pemetrexed (500 mg/m2, ivgtt 30 min) on day 1 of each 3-weeks cycle for up to six cycles. If efficacy evaluation of previous chemotherapy is progression-free (CR/PR/SD), patients will receive continuation maintenance chemotherapy (pemetrexed, 500 mg/m2, ivgtt 30 min on day 1, once every 3 weeks) until evidence of disease progression or unacceptable toxicity. All patients may also receive bevacizumab (7.5 mg/kg, ivgtt day 1, once every 3 weeks) at the investigators’ discretion.

### CHM granules

CHM Granules are composed of Hewei Granule and Feiping Granule. The formula is dynamic cycle extracted, then concentrated, and spray-dried to make granules by Jiangyin Tian Jiang Pharmaceutical Co. Ltd. (Jiangsu, China). Each package will contain water-soluble CHM Granules produced in Good Manufacture Practice (GMP) standard equipment. They were all tested by its quality management department to meet the Pharmacopoeia of China (2020 edition) grade standards. According to the Chinese Pharmacopoeia, Feiping Granule were classified into three categories based on their effects, tonifying qi (Milkvetch Root Radix Astragali, Codonopsis Radix, Atractylodis Macrocephalae Rhizoma and so on), nourishing yin (Coastal Glehnia Root, Fourleaf Ladybell Root, Cochin Chinese Asparagus Root and so on) and removing toxic substances to disintegrate a mass (Euphorbia Helioscopiae, Selaginella Doederleinii, Salviae Chinensis and so on). The detailed composition of the Hewei and Feiping formula is shown in Table 2. The methods and results of the quality control of Hewei Granule by high performance liquid chromatography (HPLC) are described in additional file 1. Our research group published a study on the identification of the main components of Feiping Granule (YiQi, YangYin and JieDu herbal medicine) by HPLC in 203 nm21, which details are available in additional file 2–4. Hewei Granule are taken orally on Days 1–7 after chemotherapy, 2 packs each time, and Feiping Granule are taken orally on Days 8–21, 2 packs each time. They are all taken with 150-200 ml of warm water twice a day after breakfast and lunch. The formula form will include an inventory list containing names and serial numbers, which will be dispensed by specific dispensing personnel to the subjects.

**Table 2:** Standard formulation of Feiping granule and Hewei granule

Pinyin name	Pharmacological name	English name	Produce from	Dosage(g)	
Feiping Fang Feiping Formula					
Huangqi	*Astragalus mongholicus* Bunge [Fabaceae]	Milkvetch Root Radix Astragali	Dry rhizoma		30
Dangshen	*Codonopsis pilosula* (Franch.) Nannf. [Campanulaceae]	Codonopsis Radix	Dry rhizoma		9
Baizhu	*Atractylodes macrocephala Koidz.* [Asteraceae]	Atractylodis Macrocephalae Rhizoma	Dry rhizoma		12
Fuling	*Poria cocos* (Schw.) Wolf. [Polyporaceae]	Indian Bread Poria	Dry sclerotia		15
Xianlingpi	*Epimedium brevicornu* Maxim. [Berberidaceae]	Epimedii Folium	Herbal		15
Huluba	*Trigonella foenum-graecum* L. [Fabaceae]	Common Fenugreek Seed Semen Trigonellae	Dry seed		15
Buguzhi	*Cullen corylifolium* (L.) Medik. [Fabaceae]	Psoralea corylifolia	Fruit		12
Beishashen	*Glehnia littoralis* (A.Gray) F.Schmidt ex Miq. [Apiaceae]	Coastal Glehnia Root	Dry rhizoma		30
Nanshashen	*Adenophora stricta Miq.* [Campanulaceae]	Fourleaf Ladybell Root	Dry rhizoma		30
Tiandong	*Asparagus cochinchinensis* (Lour.) Merr. [Asparagaceae]	Cochinchinese Asparagus Root	Dry rhizoma		15
Maidong	*Ophiopogon japonicus* (Thunb.) Ker Gawl. [Asparagaceae]	Dwarf Lilyturf Tuber	Dry rhizoma		15
Baihe	*Lilium brownii* var. viridulum Baker [Liliaceae]	Lilii Bulbus	Scale leaf		15
Nvzhenzi	*Ligustrum lucidum* W.T.Aiton [Oleaceae]	Fructus Ligustri Lucidi	Fruit		12
Xiakucao	*Prunella vulgaris* L. [Lamiaceae]	Spica Prunellae	Dry spikes		15
Shengnanxing	*Arisaema heterophyllum* Blume [Araceae]	Arisaema Rhizoma Arisaematis	Dry rhizoma		30
Sheliugu	*Amorphophallus konjac* K.Koch [Araceae]	Rhizoma Amorphophalli	Dry tubers		30
Shancigu	*Cremastra appendiculata* (D.Don) Makino [Orchidaceae]	Pseudobulbus Cremastrae Seu Pleiones	Dry pseudobulb		15
Zeqi	*Euphorbia helioscopia* L. [Euphorbiaceae]	Euphorbiae Helioscopiae	Herbal		15
Shishangbai	*Selaginella doederleinii* Hieron. [Selaginellaceae]	Selaginella Doederleinii	Herbal		30
Shijianchuan	*Salvia chinensis* Benth. [Lamiaceae]	Salviae Chinensis	Herbal		30
Chonglou	*Paris polyphylla* var. chinensis (Franch.) H.Hara [Melanthiaceae]	Rhizoma Paridis	Dry rhizoma		15
Dazao	*Ziziphus jujuba* Mill. [Rhamnaceae]	Fructus Jujubae	Fruit		9
Hewei Fang Hewei Formula					
Dangshen	*Codonopsis pilosula* (Franch.) Nannf. [Campanulaceae]	Codonopsis Radix	Dry rhizoma		15
Baizhu	*Atractylodes macrocephala Koidz.* [Asteraceae]	Atractylodis Macrocephalae Rhizoma	Dry rhizoma		12
Fuling	*Poria cocos* (Schw.) Wolf. [Polyporaceae]	Indian Bread Poria	Dry sclerotia		15
Banxia	*Pinellia ternata* (Thunb.) Makino [Araceae]	Pinellia ternata	Dry tuber		9
Chenpi	*Citrus × aurantium* L. [Rutaceae]	tangerine peel	Dry flower		9
Jixueteng	*Spatholobus suberectus Dunn* [Fabaceae]	Spatholobus suberctu	Dry lianoid stem		30
Shiwei	*Pyrrosia lingua* (Thunb.) Farw. [Polypodiaceae]	pyrrosia lingua	Dry leaf		30
Dazao	*Ziziphus jujuba* Mill. [Rhamnaceae]	Fructus Jujubae	Fruit		15
Shanzha	*Crataegus monogyna* Jacq. [Rosaceae]	hawthorn	Fruit		15
Shenqu	Massa Medicata Fermentata*	medicated leaven	Fermentata		15
Chaoguya	*Oryza sativa L.* [Poaceae]	rice malt	Seed		15
Maiya	*Hordeum vulgare* L. [Poaceae]	malt culms	Seed		15
Xianhecao	*Agrimonia pilosa Ledeb.* [Rosaceae]	Agrimonia pilosa	Dry aerial parts		30
Gancao	*Glycyrrhiza glabra* L. [Fabaceae]	radix glycyrrhizae	Dry root and rhizome		6

*Shenqu is fermented from 6 Chinese herbs, including Triticum aestivum L. [Poaceae], Prunus armeniaca L. [Rosaceae], Vigna umbellata (Thunb.) Ohwi %26 H.Ohashi [Fabaceae], Artemisia annua L. [Asteraceae], Xanthium strumarium subsp. strumarium [Asteraceae], Capsicum annuum L. [Solanaceae]

### Placebo

The placebo granules are manufactured by Jiangyin Tian Jiang Pharmaceutical Co. Ltd. (Jiangsu, China) and comply with Good Clinical Practice (GCP). They are made from food colour and artificial flavours to achieve the same appearance, colour, shape, size, smell, taste, texture and packaging as those of drugs. Ingredient (placebo) including Maltodextrin (35.34%), citric yellow pigment (13.05%), sunset yellow pigment (10.61%), caramel pigment (27.99%), bitterness (3.0%) and lactose (10.00%). All placebo granules are taken orally with 200 ml of warm water twice a day after breakfast and lunch, 2 packs each time after chemotherapy.

The study drugs are sourced from the same batch, purchased from Jiangyin Tian Jiang Pharmaceutical Co. Ltd. (Jiangsu, China). They have been securely encoded and stored in a designated cabinet by the drug administrator, ensuring room temperature and a dry environment. On the day of enrollment, patients will be provided with these medications.

Participants who show any adverse effects will be reported and followed up by their responsible physicians. A reduction in dose or discontinuation of treatment will be decided by the physician according to the NCCN Clinical Practice Guidelines in Oncology: Lung Cancer (Version 2. 2020), or upon participant request. Outcome data will be collected as usual according to the protocol for these participants.

### Outcome measurement

Tumour measurements will be performed using dynamic computed tomography (CT) or magnetic resonance imaging (MRI) at baseline and once every 2 months after treatment until disease progression or death.

### Primary outcome

#### Progression-free survival (PFS)

Defined as the time from enrollment to any documented tumour progression or death. The patients were followed up once every 2 months until death or at the end of this study to observe the tumour progression and death time.

### Secondary outcomes

#### Overall survival (OS)

Time from randomization to death due to any cause. For subjects who are lost to follow-up prior to death, the time of last follow-up will generally be calculated as the time of death.

#### Objective response rate (ORR)

Based on the revised version of the RECIST 1.1 [25] established by the National Cancer Institute (NCI), complete response (CR) and partial response (PR) are regarded as objective remission rate (ORR), ORR is calculated as CR + PR/total cases.

#### Disease control rate (DCR)

Based on the RECIST 1.1 [25], CR, PR and stable disease (SD) are regarded as DCR. DCR is calculated as CR + PR + SD/total cases.

#### Quality of life (QoL)

1. Changes in total score on the Functional Assessment of Cancer Therapy-Lung (FACT-L) questionnaire: The changes in FACT-L score will be compared in the two groups at baseline, and every 2 months after treatment until the end of this study or death. It is a universal scale for all patients with lung cancer with a total score of 108, ranging from 0 (least severe) to 108 (most severe), which consists of 36 items in 5 domains.
2. Changes in total score on the lung cancer symptom scale (LCSS): The effect of the treatment regimen on the QoL will also be evaluated by comparing the changes in the LCSS scale values at baseline and every 2 months after treatment until the end of this study or death. The scale evaluates six major symptoms associated with lung malignancies and their effects on overall symptomatic distress, functional activities, and global QOL, which has a total of 9 items, each with a score of 0–10, leading to a total score of 0–90.

#### TCM clinical syndrome scores

TCM clinical syndrome scores refer to the primary lung cancer symptom grading and quantification table in the *Guidelines for Clinical Research of New Drugs of Traditional Chinese Medicine in the Treatment of Primary Bronchogenic Cancer* (2002 Edition) issued by China State Drug Administration. The efficacy is classified into significant improvement, partial improvement, no improvement, and exacerbation: Significant improvement: ≥70% reduction in TCM clinical score, Partial improvement: ≥30% decrease in TCM clinical score, No improvement: no change in TCM clinical score, or change < 30%, Exacerbation: ≥30% increase in TCM clinical score. The calculation formula is based on the nimodipine method: Efficacy index = [(score before treatment– score after treatment) / score before treatment] × 100%. The evaluation will be performed at the time point of image evaluation.

### Safety outcomes

1. Prevalence of abnormal vital signs (pulse, respiration, blood pressure and body temperature). Measurement will be performed at baseline and every 2 months of follow-up until death or termination of the study.
2. Abnormal blood routine, urine routine, stool routine, and liver and kidney function will be examined regularly to evaluate the safety of CHM according to common terminology criteria for adverse events version 5.0 (CTCAE Version 5.0). Measurement will be performed at baseline and every 2 months during treatment, and performed by the guidelines and clinician recommendations after medication intervention.
3. Other AEs and SAEs. The Adverse Event Form will be filled out at each treatment appointment to record and grade AEs, SAEs, and abnormal laboratory results. In addition, the relationship between the AEs and CHM Granules (Hewei and Feiping Granule) should be evaluated, and treatment processes and outcomes should be documented during this period. To protect the safety of patients, immediate measures should be taken when a SAE occurs. Researchers must submit the SAE Report Form within 24 h to the research centre, medical ethics committee, and primary sponsor.

### Quality control

Researchers must be trained in good clinical practice to have the expertise, qualifications and ability to participate in clinical trials. Before the start of the project, all medical staff will be uniformly trained so that they have a full understanding of the clinical trial. Each researcher will be asked to have a resume of the investigator for easy access. Quality control of all data shall be performed once every 3 months, and the supervisory inspection problems shall be rectified within 48 h after the end of quality control.

### Data collection and monitoring

Researchers at each site will collect their data using paper CRF. Data will be collected in a dedicated database created by the Shanghai Clinical Research Center (SCRC) and will be entered and proofread independently by two data managers. The experts will check the data at each location every month and any errors found must be corrected within one week by the relevant researchers. Clinical trial researchers are the only ones who are permitted to access and keep confidential the medical records of their patients. When data processing takes place, personal information about the identifiable subject will be omitted anonymously.

It is recommended that participants visit the researchers in outpatient settings every 2 months after treatment. However, they may choose a flexible visiting time or select a follow-up method such as WeChat or telephone. To ensure that participants receive qualified medical care, the research group will provide personalised telephone and online counselling as part of the follow-up period. If they feel uncomfortable, they can contact the researchers immediately. The program will also provide incentives to help patients adhere (e.g. transportation allowances during follow-up, priority admission for lung cancer complications).

### Biological specimen collections

During the clinical study, the blood and faces will be collected from all participants with written informed consent at the time point of image evaluation, respectively. All samples will be delivered to the laboratory and stored at − 80 °C for the genetic analysis of collected blood samples and intestinal microecological analysis of collected faecal samples.

### Statistical analysis

After collection, all clinical data will be input into the computer and used in the database for all data entry. After completion, the SAS 9.0 for Windows statistical software package will be applied for statistical processing. All analyses are based on the two-sided test, with P values < 0.05 considered as statistically significant. For measurement data, the means of each group before and after treatment are compared with the paired t-test and the mutual comparison of the means between the two groups is performed with the independent sample t-test. The P value is selected according to the results of the homogeneity test of variance, and the rank sum test between the two groups is used for those who did not meet the normal distribution. For enumeration data, differences between disorder data are analyzed using Chi-squared tests, and comparisons across ordinal data are made using the Wilcoxon rank-sum test. The survival curves for PFS and OS will be estimated by the Kaplan-Meier method and will be compared between the two groups by the log-rank test. The criterion of statistical significance is 0.05. Two datasets will be generated for subsequent analysis: the intent-to-treat (ITT) population which includes all eligible participants and the per-protocol (PP) population which includes only participants who completed the trial.

### Patient and public involvement

Neither patients nor the public participate in the design, conduct, reporting, or dissemination plans of this protocol. This study will disseminate its results via telephone to study participants.

## Discussion

In recent years, with the rapid advancement of precision diagnosis and treatment for lung cancer, EGFR-TKIs have become the standard treatment for patients with EGFR-mutated advanced NSCLC. However, resistance to EGFR-TKIs remains an intractable clinical and scientific challenge. It is currently believed that secondary mutations in EGFR are the most common cause of resistance to EGFR-TKIs, with over 50% of patients found to have a T790M mutation in the EGFR gene at the time of progression on first- and second-generation TKIs [26]. In addition to T790M, there are other resistance mechanisms, including EGFR-dependent resistances such as C797X mutations, exon 20 insertion mutations, and EGFR amplifications, as well as EGFR-independent resistances like MET and HER2 amplifications, BRAF and KRAS mutations, histological transformations, etc [7]. The application status for most of these drugs developed for these mechanisms is still in its infancy and remains exploratory [27, 28].

These findings emphasize the complexity and heterogeneity of resistance mechanisms in EGFR-TKI therapy and highlight the urgent need for novel therapeutic strategies. Although third-generation EGFR-TKIs, such as Osimertinib, have provided effective treatment options for patients with the T790M mutation, resistance inevitably occurs after treatment with these agents, limiting the long-term survival of patients with advanced EGFR-mutated lung cancer [29]. Therefore, a deeper understanding of resistance mechanisms and the exploration of new treatment strategies are crucial for improving the therapeutic efficacy of EGFR-TKIs and the prognosis of patients.

In response to EGFR-TKI resistance, the clinic currently makes treatment choices based on the different patterns of disease progression in patients. When resistance appears, consolidative local ablative therapy plus continuation of EGFR-TKIs is a feasible option for patients with limited progression [30]. And for patients with systemic multiple lesions, repeated tumour biopsy is proposed in patients with resistance to EGFR-TKIs, which can analyze the possibility of resistance to EGFR-TKIs in order to select the most effective treatment, such as the third-generation EGFR-TKIs, which are recommended for patients who develop T790M. While for patients resistant to EGFR-TKIs, especially those who rapidly progress without T790M or after Osimertinib failure, platinum-based doublet chemotherapy remains the recommended regimen in the guidelines [31]. Numerous clinical trials have proven the value of pemetrexed in maintenance therapy [15, 32–34]. Phase III clinical trials have demonstrated the benefit of pemetrexed maintenance relative to placebo after platinum-based chemotherapy induction [35, 36]. Recent studies [16] have also associated bevacizumab–pemetrexed treatment with improved outcomes for patients with advanced NSCLC. However, after resistance to first-line EGFR-TKIs, conventional chemotherapy as a second-line intervention has demonstrated limited clinical efficacy, with a mPFS of only 5.4 months. In addition, the studies on the efficacy and safety of combining chemotherapy with immunotherapy are still under discussion [37, 38].

Chemotherapy, while providing limited efficacy, also induces certain side effects, including symptoms like loss of appetite, nausea, dizziness, and fatigue [39], which are recognized in TCM as spleen-stomach disharmony. Furthermore, advanced NSCLC patients often present with the syndrome of dual deficiency of qi and yin post-chemotherapy. These have been corroborated in our previous studies [40]. Our granules, as a kind of TCM preparation, based on TCM theory that mainly regulating spleen and stomach, strengthening Qi, nourishing Yin and detoxifying, possess the advantage of decreasing the side effects of chemotherapy and anti-tumour.

In preliminary basic research, we discovered that Feiping Granule exert their antitumor activity through EGR1 activation [21]. Furthermore, these granules exert their antitumor effects by alleviating T cell exhaustion and modulating the tumor microenvironment, targeting key molecular pathways and biological processes associated with lung cancer [41]. Based on these results, a previous multi-centre, double-blind, placebo-controlled clinical trial [22] CATLA study showed that first-generation EGFR-TKI combined with Feiping Granule significantly prolonged PFS (13.5 months vs. 10.94 months, *P* = 0.0064) and improved the ORR (64.32% vs. 52.66%, *P* = 0.026) and QoL in patients with stage IIIa–IV with advanced pulmonary adenocarcinoma. Adjuvant chemotherapy (vinorelbine plus cisplatin/carboplatin) combined with TCM (Feiping Granule) after radical surgery can reduce side effects and improve the temporary negative effects of adjuvant chemotherapy on QoL in patients with lung adenocarcinoma [20]. A previous study [19] also revealed that the TCM-combined therapy was numerically superior to chemotherapy alone for PFS (6.43 months VS 3.73 months, *P* = 0.019) in patients with advanced NSCLC after first-line chemotherapy. Given the aforementioned research findings, we have compelling reasons to believe that the combination of chemotherapy and TCM will have positive efficacy for patients with resistance to EGFR-TKIs. However, for patients with EGFR-TKI resistance, there is a lack of high-quality trials to demonstrate its efficacy and safety. To ensure an evidence-based approach to using Chinese herbal medicine (CHM), we have designed a randomized, double-blind, placebo-controlled multicenter clinical trial following the Standard Protocol Items: Recommendations for Interventional Trials guidelines [24, 42]. Although this protocol employs rigorous inclusion and exclusion criteria to enhance baseline homogeneity and facilitate quality control in assessing the effectiveness of CHM, potential limitations of the study should also be considered. Due to the heterogeneity of tumour cells, the resistance mechanisms substantially differ according to the specific EGFR-TKIs used [43]. We currently lack analyses of drug resistance mechanisms in patients using different EGFR-TKIs, which needs to be further refined.

There are many problems in chemotherapy after EGFR-TKIs resistance, which need to be solved urgently. Recently, Chinese medicine has always been the research hotspot in the study of lung cancer prevention and treatment. As of today, this has been the first and largest clinical trial of evidence-based medicine which uses the evaluation system of integrated traditional Chinese and Western medicine to evaluate the efficacy of Chinese herbal medicine combined with chemotherapy in the treatment of lung adenocarcinoma patients with EGFR-TKIs resistance. In this protocol, a double-blind, randomized, placebo-controlled trial will be strictly implemented in multiple research centres. As the gold standard for evaluating drug efficacy, the implementation of the RCT will provide a high-quality clinical trial methodology to prove whether TCM combined with chemotherapy treatment can prolong survival and improve quality of life among patients resistant to EGFR-targeted therapies.

## Conclusion

This study is a multicenter, blind, randomized, placebo-controlled trial that potentially provides an effective treatment strategy for the integration of traditional Chinese and Western medicine in the treatment of lung cancer.

## Electronic supplementary material

Below is the link to the electronic supplementary material.

Supplementary Material 1

Supplementary Material 2

Supplementary Material 3

Supplementary Material 4

Supplementary Material 5

Supplementary Material 6

Supplementary Material 7

Supplementary Material 8

Supplementary Material 9

## Data Availability

No datasets were generated or analysed during the current study.

## Declarations

### Trial status

This study is currently in the recruitment phase. Recruitment started in February 2021 and is expected to finish in the middle of 2024.

## Abbreviations

AC/AP: Carboplatin/Cisplatin Plus Pemetrexed
AEs: Adverse Events
ANC: Absolute Neutrophil Count
AP: Alkaline Phosphatase
ARMS: Amplification Refractory Mutation System
AST: Aspartate Aminotransferase
Bev: Bevacizumab
CHM: Chinese Herbal Medicine
CNS: Central Nervous System
CR: Complete Response
CRF: Case Report Form
CSCO: Chinese Society Of Clinical Oncology
CT: Computed Tomography
CTCAE: Common Terminology Criteria for Adverse Events
DCR: Disease Control Rate
ECOG PS: Eastern Cooperative Oncology Group Performance Status
EGFR: Epidermal Growth Factor Receptor
EGFR-TKIs: Epidermal Growth Factor Receptor-Tyrosine Kinase Inhibitors
FACT-L: Functional Assessment Of Cancer Therapy-Lung Cancer
GCP: Good Clinical Practice
GMP: Good Manufacture Practice
HPLC: High Performance Liquid Chromatography
ITT: Intent-To-Treat
LCSS: Lung Cancer Symptom Scale
LAT: Local Ablative Therapy
MRI: Magnetic Resonance Imaging
NCI: National Cancer Institute
NSCLC: Non-Small-Cell Lung Cancer
ORR: Objective Response Rate
OS: Overall Survival
PFS: Progression-Free Survival
PP: Per-Protocol
PR: Partial Response
QoL: Quality Of Life
RECIST: Response Evaluation Criteria In Solid Tumours
SAE: Serious Adverse Event
SCRC: Shanghai Clinical Research Center
SD: Stable Disease
TCM: Traditional Chinese Medicine
TKIs: Tyrosine Kinase Inhibitors
