# Supplementary material for: Efficacy and safety of Chinese herbal medicine granules plus chemotherapy in patients with EGFR-mutated advanced lung adenocarcinoma post-progression on first-line EGFR-TKI: study protocol for a multicenter, double-blind, randomized controlled trial
Source: BMC Complement Med Ther. 2025 Nov 19;25:427. doi: 10.1186/s12906-025-05037-z (PMC12628614; doi:10.1186/s12906-025-05037-z)
Supplement: Supplementary file 2 — Supplementary Material 2 [file 12906_2025_5037_MOESM2_ESM.pdf]

密　级：公开

促进市级医院临床技能与临床创新能力三年行动计划项目任务书

（2020-2022 年）

项目名称：	益气养阴解毒方联合化疗治疗 EGFR 敏感突变晚期肺腺癌的 TKI 耐药后的随机对照双盲研究
项目编号：	SHDC2020CR1052B
项目类别：	重大疾病多中心临床研究项目
计划项目类别：	重大临床研究项目
项目牵头承担单位：	上海中医药大学附属岳阳中西医结合医院（公章）
项目负责人：	许玲
执行期限：	2020 年 10 月 1 日 至 2022 年 9 月 30 日

上海申康医院发展中心

2020 年 11 月 16 日

Confidentiality Level: Public

Promoting Clinical Skills and Clinical Innovation Capacity in Municipal Hospitals

Three Year Action Plan Project Task Book

Project Name: Randomized Controlled Double-Blind Study of Qi-Yi Yang-Yin Detoxification Formula Combined with Chemotherapy for EGFR Sensitive Mutation Advanced Lung Adenocarcinoma after TKI Resistance

Project Number: SHDC2020CR1052B

Project Category: Major Disease Multicenter Clinical Research Project

Planned Project Category: Major Clinical Research Project

Leading Institution of the Project: Shanghai University of Traditional Chinese Medicine Yueyang Hospital of Integrated Traditional Chinese and Western Medicine (Official Seal)

Project Leader: Ling Xu

Execution Period: October 1, 2020 to September 30, 2022

Shanghai hospital development center

November 16, 2020
