# Supplementary material for: Efficacy and safety of Chinese herbal medicine granules plus chemotherapy in patients with EGFR-mutated advanced lung adenocarcinoma post-progression on first-line EGFR-TKI: study protocol for a multicenter, double-blind, randomized controlled trial
Source: BMC Complement Med Ther. 2025 Nov 19;25:427. doi: 10.1186/s12906-025-05037-z (PMC12628614; doi:10.1186/s12906-025-05037-z)
Supplement: Supplementary file 3 — Supplementary Material 3 [file 12906_2025_5037_MOESM3_ESM.pdf]

项目编号	类 别	建设年限	总经费（万元）
2019XZZX-ZL004	□基本中医药循证能力建设<br>■专科专病循证能力提升	2019 年 5 月--2021 年 5 月	200

中医药循证能力建设项目
任务书

项目名称 中医药治疗Ⅳ期 EGFR 突变阳性肺癌的中医肿瘤循证能力建设及循证实施方案研究

项目负责人 许玲

承担单位 上海中医药大学附属岳阳中西医结合医院

联系电话 15901903361

电子邮箱 xulq67@aliyun.com

填报日期 2019-12-16

国家中医药管理局

二〇一九年九月制

Project Number	Category	Execution Period	Total Funding (ten thousand RMB)
2019XZZX-ZL004	☐Basic Traditional Chinese Medicine (TCM) Evidence-Based Capacity Building<br>■Specialty Disease Evidence-Based Capacity Enhancement	May 2019 - May 2021	200

Task Book of Traditional Chinese Medicine Evidence-Based Capacity Construction Project

Project Name: Research on the Construction and Implementation of Evidence-Based Practice in TCM Oncology for the Treatment of Stage IV EGFR Mutation Positive Lung Cancer

Project Leader: Ling Xu

Executing Unit: Shanghai University of Traditional Chinese Medicine Yueyang Hospital of Integrated Traditional Chinese and Western Medicine

Contact Number: 15901903361

Email: xulq67@aliyun.com

Date of Submission: 2019-12-16

Issued by National Administration of Traditional Chinese Medicine

September 2019
