# Supplementary figures and images for: Efficacy and safety of Chinese herbal medicine granules plus chemotherapy in patients with EGFR-mutated advanced lung adenocarcinoma post-progression on first-line EGFR-TKI: study protocol for a multicenter, double-blind, randomized controlled trial
Source: BMC Complement Med Ther. 2025 Nov 19;25:427. doi: 10.1186/s12906-025-05037-z (PMC12628614; doi:10.1186/s12906-025-05037-z)

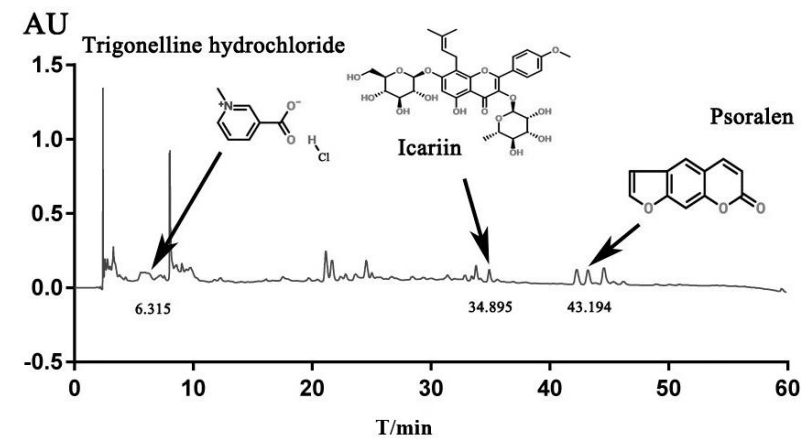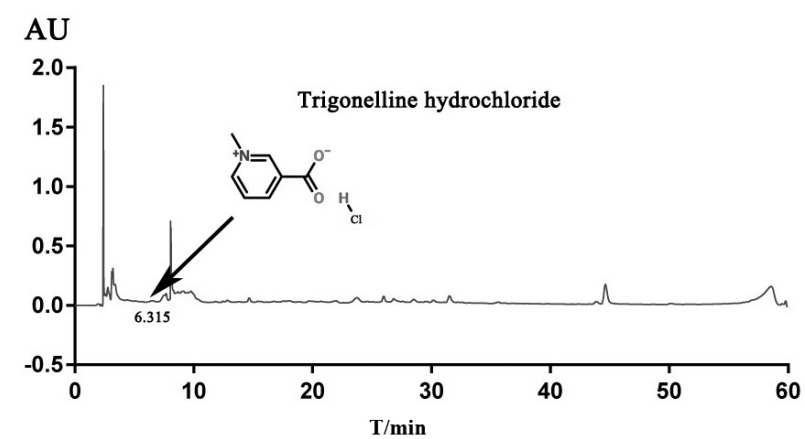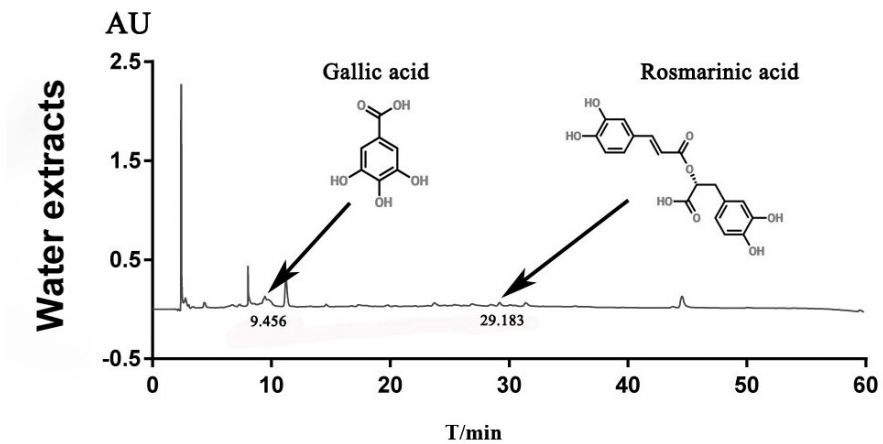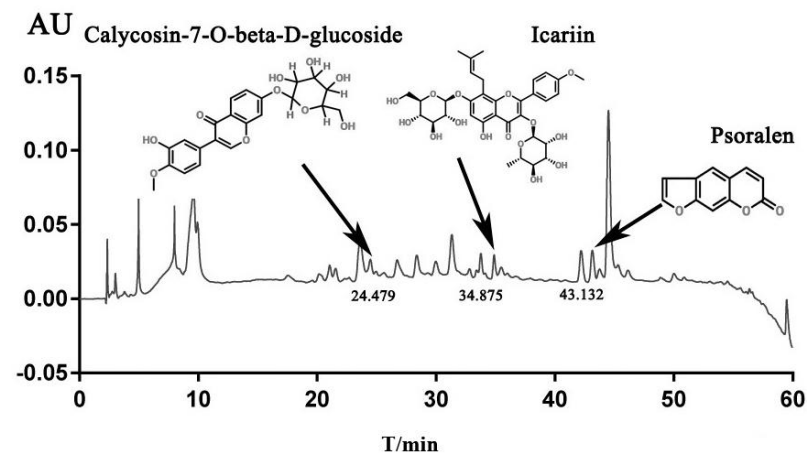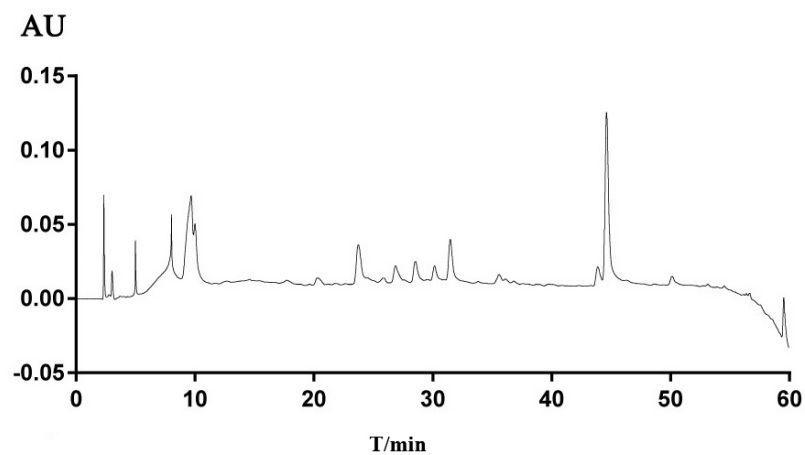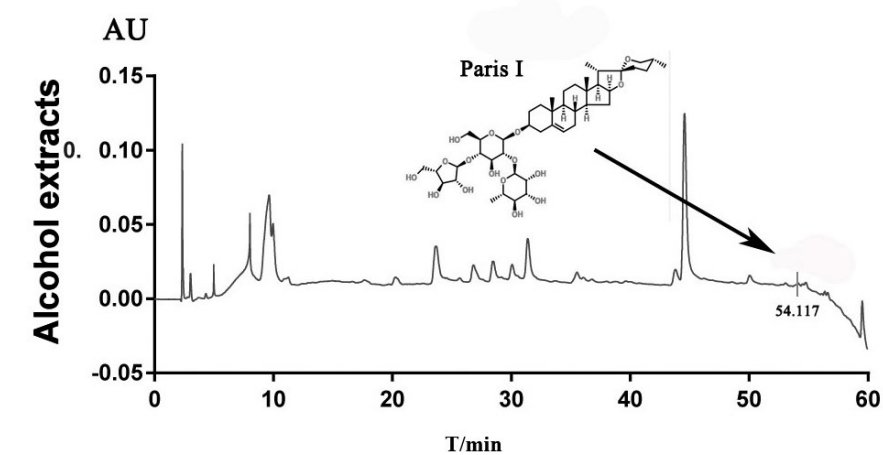

Additional file 2. The water and alcohol extracts of YiQi, YangYin and JieDu Herbal Medicine

Supplement: Supplementary file 4 — Supplementary Material 4 [file 12906_2025_5037_MOESM4_ESM.pdf]
