# Supplementary material for: Efficacy and safety of Chinese herbal medicine granules plus chemotherapy in patients with EGFR-mutated advanced lung adenocarcinoma post-progression on first-line EGFR-TKI: study protocol for a multicenter, double-blind, randomized controlled trial
Source: BMC Complement Med Ther. 2025 Nov 19;25:427. doi: 10.1186/s12906-025-05037-z (PMC12628614; doi:10.1186/s12906-025-05037-z)
Supplement: Supplementary file 5 — Supplementary Material 5 [file 12906_2025_5037_MOESM5_ESM.pdf]

课题编号：2023YFC3503302　　　　　　　　　　　　　密　级：公开

国家重点研发计划
课题任务书

课题名称：中西医结合优化方案提升肺癌靶向治疗耐药后生存获益研究

所属项目：非小细胞肺癌中西医结合防治关键技术与诊疗方案研究

所属专项：中医药现代化

项目牵头承担单位：中国中医科学院广安门医院

课题承担单位：上海中医药大学附属岳阳中西医结合医院

课题负责人：许玲

执行期限：2023年11月 至 2027年10月

中华人民共和国科学技术部制

2023年11月23日

0003YF 2023YFC3503302 2023-11-23 09:44:42

Project Number: 2023YFC3503302 **Confidentiality Level: Public**

National Key Research and Development Program

Project Task Book

Project Subject Name: Research on Optimizing Integrated Traditional Chinese and Western Medicine Schemes to Improve Survival Benefits after Targeted Therapy Resistance in Lung Cancer

Project Belongs to: Research on Key Technologies and Diagnostic and Treatment Schemes for the Prevention and Treatment of Non-Small Cell Lung Cancer with Integrated Traditional Chinese and Western Medicine

Special Program: Modernization of Traditional Chinese Medicine

Leading Institution of the Project: Guang'anmen Hospital, China Academy of Chinese Medical Sciences

Executing Institution of the Project Subject: Shanghai University of Traditional Chinese Medicine Yueyang Hospital of Integrated Traditional Chinese and Western Medicine

Project Subject Leader: Ling Xu

Execution Period: November 2023 to October 2027

Issued by the Ministry of Science and Technology of the People's Republic of China

November 23, 2023
