# Supplementary material for: Efficacy and safety of Chinese herbal medicine granules plus chemotherapy in patients with EGFR-mutated advanced lung adenocarcinoma post-progression on first-line EGFR-TKI: study protocol for a multicenter, double-blind, randomized controlled trial
Source: BMC Complement Med Ther. 2025 Nov 19;25:427. doi: 10.1186/s12906-025-05037-z (PMC12628614; doi:10.1186/s12906-025-05037-z)
Supplement: Supplementary file 6 — Supplementary Material 6 [file 12906_2025_5037_MOESM6_ESM.pdf]

伦理审查批件

批件号	2020—078
项目名称	益气养阴解毒方联合化疗治疗 EGFR 敏感突变晚期肺腺癌的 TKI 耐药后的随机对照双盲研究
申办者	上海中医药大学附属岳阳中西医结合医院
研究单位	上海中医药大学附属岳阳中西医结合医院、上海市胸科医院、上海市肺科医院、复旦大学附属肿瘤医院、上海交通大学医学院附属瑞金医院
主要研究者	许玲
审查类别及方式	初始审查（2020 年申康项目中标后）：会议审查
审查日期	2021 年 2 月 4 日
审查委员	郑　莉、王雪文、张春雁、黄　瑾、徐玲玲、冯寿全、史　晓、马晓芃、范斌、刘巍峰、孙武权、郝微微、樊民胜、任　力、姚永其
批准文件	1. 初始伦理审查申请<br>2. 研究者经济利益声明<br>3. 临床研究方案（版本号：V2.0；版本日期：2020 年 12 月 15 日）<br>4. 知情同意书（版本号：V2.0；版本日期：2020 年 12 月 15 日）<br>5. 招募广告（版本号：V2.0；版本日期：2020 年 12 月 15 日）<br>6. 病例报告表（版本号：V2.0；版本日期：2020 年 12 月 15 日）<br>7. 受试者身份识别代码表<br>8. 主要研究者简历、培训证书<br>9. 项目任务书

审查意见

根据卫健委《涉及人的生物医学研究伦理审查办法》（2016）、NMPA《药物临床试验质量管理规范》、《医疗器械临床试验管理规范（2016）》、WMA《赫尔辛基宣言》和 CIOMS《人体生物医学研究国际道德指南》的伦理原则，经本伦理委员会审查，该项目符合伦理原则。

请遵循 GCP 原则、遵循伦理委员会批准的方案开展临床研究，保护受试者的健康与权利。

研究开始前，请申请人完成临床试验注册。

研究过程中若变更主要研究者，对临床研究方案、知情同意书、招募材料等的任何修改，请申请人提交修正案审查申请。

发生严重不良事件，请申请人及时提交严重不良事件报告。

请按照伦理委员会规定的年度 / 定期跟踪审查频率，申请人在截止日期前 1 个月提交研究进展报告；申办者应当向组长单位伦理委员会提交各中心研究进展的汇总报告；当出现任何可能显著影响试验进行、或增加受试者危险的情况时，请申请人及时向伦理委员会提交书面报告。

研究纳入了不符合纳入标准或符合排除标准的受试者，符合中止试验规定而未让受试者退出研究，给予错误治疗或剂量，给予方案禁止的合并用药等没有遵从方案开展研究的情况；或可能对受试者的权益 / 健康以及研究的科学性造成不良影响等违背 GCP 原则的情况，请申办者 / 监察员 / 研究者提交违背方案报告。

申请人暂停或提前终止临床研究，请及时提交暂停 / 终止研究报告。

完成临床研究，请申请人提交研究完成报告。

扫描全能王 创建

年度/定期跟踪审查频率	口 3 个月，口 6 个月，■12 个月
批件有效期	2021 年 02 月 05 日——2022 年 02 月 04 日
联系人与联系电话	伦理委员会秘书：殷从全　联系电话：021-65161782-8122
主任委员签名	[illegible]
伦理委员会	上海中医药大学附属岳阳中西医结合医院伦理委员会（盖章）
日期	2021 年 02 月 05 日

扫描全能王 创建
