# Supplementary material for: Efficacy and safety of Chinese herbal medicine granules plus chemotherapy in patients with EGFR-mutated advanced lung adenocarcinoma post-progression on first-line EGFR-TKI: study protocol for a multicenter, double-blind, randomized controlled trial
Source: BMC Complement Med Ther. 2025 Nov 19;25:427. doi: 10.1186/s12906-025-05037-z (PMC12628614; doi:10.1186/s12906-025-05037-z)
Supplement: Supplementary file 8 — Supplementary Material 8 [file 12906_2025_5037_MOESM8_ESM.pdf]

Science and Technology Plan Project Contract

Project Number: 21MC1930500

Project Name: Shanghai Clinical Research Center of Traditional Chinese Medicine Oncology

Executing Unit: Longhua Hospital Affiliated to Shanghai University of Traditional Chinese Medicine

Project Leader: Hegen Li

Execution Period: October 1, 2021 to September 30, 2024

Issued by the Shanghai Municipal Science and Technology Commission

科技计划项目合同

项目编号： 21MC1930500

项目名称： 上海市中医肿瘤临床医学研究中心

项目承担单位： 上海中医药大学附属龙华医院

项目负责人： 李和根

项目执行期： 2021-10-01 至 2024-09-30

上海市科学技术委员会制

STCSM

委托单位（甲方）：上海市科学技术委员会

承担单位（乙方）：上海中医药大学附属龙华医院

甲乙双方根据《上海市科技计划项目管理办法（试行）》、《上海市科研计划项目（课题）专项经费管理办法》、《上海市科技计划项目综合绩效评价工作规范（试行）》等文件规定及有关法律政策，在上海市黄浦区签署本合同。签约双方一致同意诚信履行本合同约定的权利和义务，相关内容作为项目实施管理、监督和评价的重要依据。

一、项目基本情况：

签约双方一致同意本合同所附项目任务书内容，严格按照项目任务书的研究内容和考核指标实施项目。

项目编号：21MC1930500

项目名称：上海市中医肿瘤临床医学研究中心

项目承担单位：上海中医药大学附属龙华医院

项目负责人：李和根

项目执行期：2021-10-01 至 2024-09-30

二、拨款计划：

	拨（贷）款安排	金 额（千元）
项目编号：21MC1930500<br>计划拨款：5000.0 千元	首期拨款	4000.0
	中期拨款	/
	验收后拨款	1000.0

三、共同条款：

(一)项目经费管理

1、乙方应当遵守《上海市科技计划项目管理办法（试行）》和《上海市科研计划项目（课题）专项经费管理办法》。乙方若将甲方所拨资金挪作他用或在本合同生效之日起半年内仍未开展工作的，甲方将直接单方面终止该合同，乙方应全部退还甲方所拨资金。

2、乙方按规定应退回结余经费的，乙方应按照甲方要求在规定时间内退回结余经费到甲方指定账户。乙方未按照甲方要求退回结余经费的，甲方将乙方纳入科研信用记录。

(二)项目实施管理

3、甲方可委托项目管理机构承担项目实施过程管理等业务管理职能。乙方应配合甲方或甲方委托的项目管理机构开展相关项目管理工作，为其履行职责提供必要支持。

4、项目执行过程中，乙方应遵守人类遗传资源管理、实验动物管理等相关法规和规范性文件。

5、项目执行过程中，对项目取得的重大进展，以及可能影响项目实施的重大事项或问题，乙方应当及时报告甲方。

6、项目执行过程中，乙方若不能按期完成需延期的或发生需要报甲方审批的调整事项，应在项目原定结束日期3个月前提交变更申请，经甲方审批后实施。

7、乙方若不能履行合同，应提交终止合同申请。甲方或甲方委托的项目管理机构对情况进行核实确认，由甲方向乙方发出合同终止通知。

8、乙方未能按约履行合同、乙方不接受不配合甲方或甲方委托的项目管理机构对项目的监督检查且经催告后仍不配合的、乙方严重违规违纪且不按规定进行整改或拒绝整改的，甲方将直接单方面终止该合同。

9、合同因故终止，甲方或甲方委托的项目管理机构将组织清查处理，乙方应积极配合清理账目和资产，编制决算报表和资产清单上报甲方。合同终止后，乙方应将结余经费（含处理已购仪器、设备及材料的变价收入）归还甲方。对于因非正当理由致使合同撤销或终止的，甲方将负有过错的乙方或项目负责人纳入科研信用记录。

(三)项目验收

10、乙方应当遵守甲方制定发布的《上海市科技计划项目综合绩效评价工作规范（试行）》，在项目执行期结束后3个月内，提交验收申请材料，包括项目验收申请、项目综合绩效自评价报告、科技报告等。其中科技报告作为项目验收的必备材料，按照标准格式和规范撰写和提交，并依据《上海市科技计划科技报告管理办法》予以公开。

11、乙方在项目执行期结束6个月后，仍未向甲方或甲方委托的管理机构提交项目验收申请及上述验收材料的，甲方将直接单方面终止该合同。

(四)知识产权归属

12、本项目形成的知识产权，按下列第__2__项分配：

（1）归甲方所有。乙方拥有免费使用的权利。

（2）归乙方所有。为了国家安全、国家利益和重大社会公共利益的需要，甲方可以无偿实施，也可以许可他人有偿实施或者无偿实施。

(五) 资助成果标注

13、项目形成的研究成果，包括论文、专著、样机、样品、视频等，均应如实注明得到上海市科技计划项目资助和项目编号。

(六)文件资料归档

14、乙方为科研项目文件资料归档责任人。乙方应配合甲方或甲方委托的管理机构做好项目文件资料的归档工作。

15、项目文件资料的归档范围包括项目申请、立项、过程管理和验收、财务监督审计等相关工作所产生的重要文件。

(七)科普义务

16、乙方在进行项目研究过程中或完成项目研究形成成果后，有义务向公众传播和普及相关的科学知识、科学方法、科学思想、科学精神。

17、在不涉及乙方技术和商业秘密的前提下，甲方可以无偿利用项目成果开展科学普及，乙方应当予以配合。

(八)诚信义务

18、乙方在进行项目实施及相关活动中应当恪守科研诚信要求和科研伦理准则。经查实乙方或相关人员存在科研不端行为的，甲方有权将具体情形纳入科研信用记录。

四、附加条款：

STCSM

项目成果需优先在本地产业化或优先转让本市企业。

五、本合同一式 6 份。合同采用A4纸双面打印，装订成册加盖骑缝章。

六、本合同在签约各方签章后生效。

七、因履行本合同发生的争议，由当事人协商解决，协商不成的，依法向合同签署地人民法院起诉。

STCSM

本合同签约双方：

委托单位（甲方）：　上海市科学技术委员会

代　　表：＿＿＿＿＿＿＿＿（签章）

财务负责人：＿＿＿＿＿＿＿＿（签章）

（公章）　地　　址：人民大道200号（200003）

电　　话：

日　　期：　　年　　月　　日

项目承担单位（乙方）：

法人的法定代表人/非法人组织的负责人：肖臻（签章）

项目负责人：李和根（签章）

财务负责人：马月兰（签章）

地　　址：上海市宛平南路725号

电　　话：13801909427

开 户 名：上海中医药大学附属龙华医院

（公章）　开户银行：上海银行衡山支行

银行账号：316861-00005014088

日　　期：　　年　　月　　日

STCSM
