# Supplementary material for: Efficacy and safety of Chinese herbal medicine granules plus chemotherapy in patients with EGFR-mutated advanced lung adenocarcinoma post-progression on first-line EGFR-TKI: study protocol for a multicenter, double-blind, randomized controlled trial
Source: BMC Complement Med Ther. 2025 Nov 19;25:427. doi: 10.1186/s12906-025-05037-z (PMC12628614; doi:10.1186/s12906-025-05037-z)
Supplement: Supplementary file 9 — Supplementary Material 9 [file 12906_2025_5037_MOESM9_ESM.pdf]

Ethics Review Approval

**Approval Number**	2020—078
**Project Name**	A Randomized, Double-Blind Study of Qi-Boosting, Yin-Nourishing, and Toxin-Resolving Formula Combined with Chemotherapy for EGFR Sensitive Mutation Advanced Lung Adenocarcinoma with TKI Resistance
**Sponsor**	Shanghai Yueyang Hospital of Integrated Traditional Chinese and Western Medicine Affiliated to Shanghai University of Traditional Chinese Medicine
**Research Units**	Shanghai Yueyang Hospital of Integrated Traditional Chinese and Western Medicine Affiliated to Shanghai University of Traditional Chinese Medicine, Shanghai Science Hospital, Shanghai Pulmonary Hospital, Fudan University Shanghai Cancer Center, Ruijin Hospital Affiliated to Shanghai Jiao Tong University School of Medicine
**Principal Investigator**	Xu Ling
**Review Category and Method**	Initial Review (after winning the 2020 Shenkang Project): Meeting Review
**Review Date**	February 4, 2021
**Review Committee Members**	Zheng Li, Wang Xuewen, Zhang Chunyan, Huang Jin, Xu Lingling, Feng Shouquan, Shi Xiao, Ma Xiaopeng, Fan Bin, Liu Weifeng, Sun Wuquan, Hao Weiwei, Fan Minsheng, Ren Li, Yao Yongqi
**Approved Documents**	1. Initial Ethics Review Application<br>2. Investigator's Financial Interest Statement<br>3. Clinical Research Protocol (Version Number: V2.0; Version Date: December 15, 2020)<br>4. Informed Consent Form (Version Number: V2.0; Version Date: December 15, 2020)<br>5. Recruitment Advertisement (Version Number: V2.0; Version Date: December 15, 2020)<br>6. Case Report Form (Version Number: V2.0; Version Date: December 15, 2020)<br>7. Subject Identification Code Table<br>8. Principal Investigator's Resume and Training Certificate<br>9. Project Task Book
Review Opinion	
In accordance with the ethical principles of the National Health Commission's "Measures for the Ethical Review of Biomedical Research Involving Human Beings" (2016), NMPA's "Good Clinical Practice," "Good Clinical Practice for Medical Devices" (2016), WMA's "Declaration of Helsinki," and CIOMS's "International Ethical Guidelines for Biomedical Research Involving Human Subjects," the project has been reviewed by the Ethics Committee and is considered to comply with ethical principles.<br>Please follow the GCP principles and conduct the clinical research in accordance with the approved protocol by the Ethics Committee to protect the health and rights of the subjects.<br>Before the start of the research, please complete the clinical trial registration.<br>If there is a change in the principal investigator, or any modifications to the clinical research protocol, informed consent form, recruitment materials, etc., please submit a protocol amendment review application.	

<table>
<tr><td colspan="2">In the event of a serious adverse event, please submit a serious adverse event report in a timely manner.
Please submit a study progress report one month before the deadline according to the annual/regular follow-up review frequency specified by the Ethics Committee: The sponsor should submit a summary report of the study progress from each center to the Ethics Committee of the lead unit; if any situation occurs that may significantly affect the trial or increase the risk to the subjects, please submit a written report to the Ethics Committee in a timely manner.
If there are any violations of the protocol, such as including subjects who do not meet the inclusion criteria or meet the exclusion criteria, failing to withdraw subjects from the study in accordance with the termination regulations, administering incorrect treatment or dosage, or using prohibited concomitant medications specified in the protocol, which may adversely affect the rights/health of the subjects or the scientific nature of the study, please submit a protocol deviation report by the sponsor/monitor/investigator.
If the applicant suspends or terminates the clinical research prematurely, please submit a suspension/termination report in a timely manner.
Upon completion of the clinical research, please submit a final study report.</td></tr>
<tr><td>Annual/Regular Follow-up Review Frequency</td><td>[ ] Every 3 months, [ ] Every 6 months, [√] Every 12 months</td></tr>
<tr><td>Approval Period</td><td>February 5, 2021 – February 4, 2022</td></tr>
<tr><td>Contact Person and Telephonec</td><td>Ethics Committee Secretary: Yin Congquan, Telephone: 021-65161782-8122</td></tr>
<tr><td>Signature of the Chairperson</td><td>Zheng Li</td></tr>
<tr><td>Ethics Committee</td><td>Ethics Committee of Shanghai Yueyang Hospital of Integrated Traditional Chinese and Western Medicine Affiliated to Shanghai University of Traditional Chinese Medicine (Seal)</td></tr>
<tr><td>Date</td><td>February 5, 2021</td></tr>
</table>
