# Supplementary material for: Efficacy and safety of Chinese herbal medicine granules plus chemotherapy in patients with EGFR-mutated advanced lung adenocarcinoma post-progression on first-line EGFR-TKI: study protocol for a multicenter, double-blind, randomized controlled trial
Source: BMC Complement Med Ther. 2025 Nov 19;25:427. doi: 10.1186/s12906-025-05037-z (PMC12628614; doi:10.1186/s12906-025-05037-z)
Supplement: Supplementary file 7 — Supplementary Material 7 [file 12906_2025_5037_MOESM7_ESM.pdf]

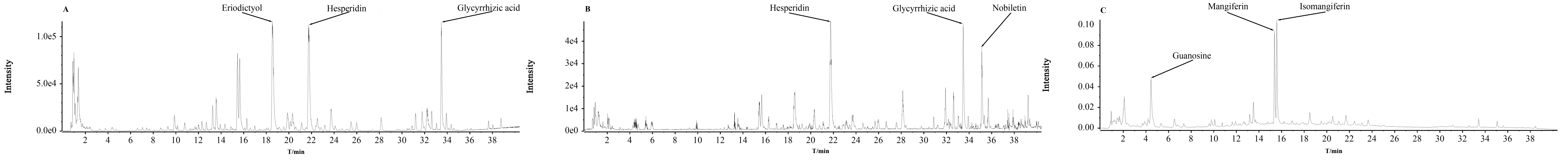

Fig S1. Results of UPLC-Q-TOF-MS analysis of Hewei Granules. A The Base Peak Chromatogram (BPC) of Hewei Granules in negative ion mode. B Base Peak Chromatogram (BPC) of Hewei Granules in positive ion mode. C UPLC UV chromatogram of Hewei Granules sample-254 nm.
